# High mobility group box- 1 levels may be associated with disease activity of Behcet’s disease

**DOI:** 10.3906/sag-2101-116

**Published:** 2021-10-21

**Authors:** Dilara DÖNMEZ GÜLER, Ayşe Bahar KELEŞOĞLU DİNÇER, Zeynep Ceren KARAHAN, Hasan Selim GÜLER, Müçteba Enes YAYLA, Serdar SEZER, Emine Gözde AYDEMİR GÜLÖKSÜZ, İlyas Ercan OKATAN, Murat TORGUTALP, Didem ŞAHİN EROĞLU, Mehmet Levent YÜKSEL, Tahsin Murat TURGAY, Gülay KINIKLI, Aşkın ATEŞ

**Affiliations:** 1 Department of Internal Medical Sciences, Faculty of Medicine, Ankara University, Ankara Turkey; 2 Division of Rheumatology, Department of Internal Medicine, Faculty of Medicine,Ankara University, Ankara Turkey; 3 Department of Medical Microbiology, Faculty of Medicine, Ankara University, Ankara Turkey

**Keywords:** High mobility group box- 1, autoimmune diseases, Behcet’s disease, inflammation

## Abstract

**Background/aim:**

High mobility group box- 1 (HMGB- 1) is a nuclear protein acting as a proinflammatory molecule. The serum HMGB- 1 levels were found elevated in chronic inflammatory diseases. In this cross-sectional study, serum HMGB- 1 levels in Behcet’s disease (BD) patients and healthy controls (HC) were studied. Also, its association with disease activity scores and clinical findings were evaluated.

**Materials and methods:**

Ninety BD patients and 50 age-sex matched HC were included in the study. Disease activity scores were assessed by Behcet Disease Current Activity Form (BDCAF) and Behcet Syndrome Activity Score (BSAS). Serum HMGB- 1 levels were measured using a commercial ELISA kit. A *p* value of < 0.05 was considered to be statistically significant.

**Results:**

Serum HMGB- 1 levels were significantly higher in BD than in HC (43.26 pg/mL and 16.73 pg/mL; *p *< 0.001, respectively). Serum HMGB- 1 levels were statistically significantly associated with presence of erythema nodosum (EN) and genital ulcers in the last one month prior to recruitment (*p *= 0.041 and *p *< 0.001, respectively). BDCAF and BSAS scores were positively correlated with serum HMGB- 1 level ( *p *= 0.03 and *p *= 0.02, respectively).

**Conclusion:**

HMGB - 1 may play a role in the development of BD. Also, due to its positive correlation with disease activity indices, it can be used as a novel disease activity parameter in BD.

## 1. Introduction

Behcet’s disease is a chronic inflammatory disease characterized by various systemic symptoms including skin lesions such as recurrent oral ulcers, genital ulcers, EN and papulopustular eruption (PPE), eye involvement such as uveitis and retinal vasculitis, musculoskeletal manifestations such as arthritis and arthralgia, and gastrointestinal, neurological, vascular, cardiac and pulmonary involvements [1]. Although its etiopathogenesis is not fully known, genetic factors and immune dysregulation are considered to play a significant role in the development of this disease [2].

High mobility group box- 1 (HMGB- 1) is a nonhistone protein with important biological activities both inside and outside the cell. HMGB- 1 is comprised of 215 amino acids, has a molecular weight of ~ 25 kDa, and includes two DNA-binding domains (A and B boxes) and a C-terminal acidic tail [3]. HMGB- 1 binds to different molecules through different receptors, leading to different metabolic functions. Receptor for Advance Glycation End Product (RAGE), a transmembrane protein belonging to the immunoglobulin (Ig) superfamily, is the first receptor described for HMGB- 1 [4]. Binding of HMGB- 1 with RAGE causes cell migration [5], whereas binding with Toll-like receptor 4 (TLR4) mediates inflammation through NF-κB activation and production of cytokines such as interleukin- 6 (IL- 6) and tumor necrosis factor-α (TNF-α) by macrophages [6-8], and binding to p53 causes gene transcription [9]. Extracellular HMGB- 1 arouses a potent inflammatory response stimulating the release of proinflammatory cytokines in neutrophils and macrophages [10]. Elevated serum HMGB- 1 level is reported in many autoimmune and inflammatory diseases [11–13]. 

Neutrophilic infiltration, endothelial cell swelling, necrosis and elevated serum levels of cytokines that prime the neutrophils were shown in the pathogenesis of BD [14]. These findings suggested BD to be a neutrophilic vasculitis [15]. Activated neutrophils in BD secrete cytokines that both prime themselves and stimulate T-helper- 1 cells [14]. Emmi G and his collegeues demonstrated the important role of neutrophils in the development of thrombotic events in BD. They showed an increased leucocyte oxidative stress and radical oxygen species leading to posttranslational carbonylation of fibrinogen and reduced plasmin lysis, which were thought to play role in thrombus formation in BD [16]. On the other hand, intensive leucocytes recruitment is the most significant feature of HMGB- 1 associated inflammation [17]. HMGB- 1 can directly activate neutrophils to produce IL- 1β, IL- 8 and TNF-α, which are one of the main cytokines of inflammation and related diseases [18]. With respect to this knowledge, we aimed to evaluate the role of HMGB- 1 in BD and its association with clinical manifestations and disease activity. 

## 2. Materials and methods

### 2.1. Study population

Ninety BD patients over the age of 18 who were diagnosed according to the 1990 International Study Group criteria for Behcet’s Disease [19] and followed between March 2018 and March 2019 at Ankara University Faculty of Medicine, Ibn-i Sina Hospital, Behcet’s Disease Multidisciplinary Diagnosis and Treatment Unit, and 50 age and sex compatible HC were recruited consequtively for the study. Patients with autoimmune or autoinflammatory diseases other than BD and patients with other comorbidities or infectious diseases during recruitment were excluded from the study.

### 2.2. Study design

Demographic data, clinical and treatment features of BD patients were obtained from patients at the time of study recruitment and also from the hospital records. Mucocutaneous manifestations included recurrent aphthous stomatitis, genital ulcers, EN, PPE and skin pathergy reaction. Papulopustular eruptions were defined as folliculitis or acne like lesions with sterile erythematous papules or pustules that are localized mostly on the lower extremities but also on upper extremities, trunk, face and neck [20, 21]. Mucocutanous involvement was assessed by physical examination at the time of recruitment. Joint involvement was described as synovial inflammation which could not be otherwise explained by any other inflammatory arthritis and was diagnosed by physical examination and imaging studies as conventional radiography, ultrasound and/or magnetic resonance imaging (MRI). Ocular involvement included inflammation of uvea and retina (namely, uveitis and retinis, respectively). Uveitis was classified as isolated anterior uveitis, posterior uveitis and panuveitis according to ophthalmologist’s examination. Vascular involvement included both arterial and venous involvements. Pulmonary and/or peripheral artery aneurisms and/or thrombosis were classified as arterial involvement; whereas deep venous thromboses were classified as venous involvement. The diagnosis of vascular involvement was confirmed by arterial and/or venous doppler ultrasonography, computed tomography, MRI, echocardiography and/or scintigraphy. Gastrointestinal system (GIS) involvement was defined as presence of punched-out or aphthous ulcers in endoscopic studies in patients with gastrointestinal symptoms that could not be explained by other diseases. Central nervous system (CNS) involvement was defined as presence of parenchymal disease and/or cerebral venous sinus thrombosis in neuroimaging studies in patients with neurological symptoms that could not be explained by another systemic/neurological disease or usage of any drug. Major organ involvement (MOI) was defined as the presence of at least one of the eye, vascular, CNS or GIS involvements as described previously. 

The disease activity of BD was evaluated at hospital visit during recruitment. In order to evaluate disease activity; two measures were used. One of the measures used was Behcet Syndrome Activity Score (BSAS) which is a ten-question patient derived assessment tool. It scores the degree of discomfort caused by the current clinical findings over the last four weeks of patient assessment by the patient visual analogue scales [22, 23]. It is scored between 0 - 100. The other activity index used was Behcet Disease Current Activity Form (BDCAF) which is a commonly used index that scores the absence or presence of clinical manifestations over the last four weeks prior to patient hospital visit [23, 24]. The total score is 12. Patients with a score of ≥ 2 were accepted as “active” [24, 25]. Both indices had been translated to Turkish correctly and are commonly used in daily practice [23]. 

Peripheral blood samples were drawn from BD patients for routine evaluation of Erythrocyte Sedimentation Rate (ESR) (0 - 20 mm/h), C-Reactive protein (CRP) (0 - 5 mg/L) and complete blood count (CBC). 

### 2.3. Enzyme-linked immunosorbent assay (ELISA)

Peripheral venous blood samples were collected from BD patients and HC by using proper tubes to determine their serum HMGB- 1 levels, and then the samples were centrifuged at 3000 rpm for 15 minutes and stored at - 80 °C. The samples were processed using ELISA kits (LifeSpan BioSciences, Inc., Seattle, USA) (Catalog No: LS-F11641) at Ankara University, Faculty of Medicine, Ibn-i Sina Hospital Microbiology Laboratory.

### 2.4. Ethical considerations

The study was conducted according to the Declaration of Helsinki and written consent was received from patients. The study protocol was approved by Ankara University Faculty of Medicine Ethics Board (Approval Number: 12 - 724 - 17).

### 2.5 Statistical analysis

Data were statistically analyzed using the IBM SPSS for Windows Version 22.0 (SPSS, Chicago, USA). The normality of numerical variables was examined with the Kolmogorov Smirnov test. If numerical variables had normal distribution, they were shown by mean ± standard deviation (SD), otherwise by median (minimum and maximum) values. Categorical variables were shown using numbers and percentages. The comparison between healthy control and BD patients’ demographic data was examined using the t test when the variables were normally distributed and using the Mann–Whitney U test when they were not normally distributed. The relationship of BD patients’ clinical findings with HMGB- 1 levels was examined using the Mann–Whitney U test. Power analyses were performed using G^*^Power version 3.0.10 for sample size calculation. Statistically significance level was considered as *p* < 0.05.

### 2.6. Funding

The study was funded by the Scientific Research Projects Unit of Ankara University (Project number: 18L0230003).

## 3. Results

### 3.1. Demographic and clinical features 

The demographic and clinical features of BD patients were summarized in Table 1. Median serum HMGB- 1 levels were significantly higher in BD patients (43.26 (0 – 221.33) pg/mL) in comparison to HC (16.73 (0– 41.95) pg/mL) (*p *< 0.001). 

**Table 1:** Demographic, clinical characteristics since disease onset and laboratory findings of BD patients.

	Behcet’s disease	Healthy control	p value
Age, years, mean (± SD)	42.16 ± 9.69	39.00 ± 10.65	0.128
Sex, women, n (%)	51 (56.70)	32 (64.00)	0.401
Disease duration (years), median (min - max)	10 (1–37)		
Clinical manifestations, n (%)			
Oral ulcers	90 (100.00)		
Genital ulcers	68 (75.55)		
Erythema nodosum like lesions	52 (57.77)		
Papulopustuler eruption	59 (65.55)		
Pathergy positivity	37 (41.11)		
Uveitis	27 (30.00)		
Retinal vasculitis	2 (2.22)		
CNS involvement	7 (7.77)		
GIS involvement	2 (2.22)		
Arthralgia	75 (83.33)		
Arthritis	15 (16.66)		
Arterial involvement	3 (3.33)		
Venous involvement	25 (27.77)		
Major organ involvement	47 (52.22)		
Treatment, n (%)			
Colchicine	86 (95.55)		
Azathioprine	20 (22.22)		
Mycophenolate mofetil	1 (1.11)		
Methotrexate	1 (1.11)		
Cyclophosphamide	1 (1.11)		
Sulfasalazine	2 (2.22)		
Adalimumab	2 (2.22)		
Laboratory findings, median (min - max)			
Serum HMGB- 1 level (pg/mL)	43.26 (0 –221.33)	16.73 (0– 41.95)	< 0.001*
ESR (mm/h)	9 (1– 48)		
CRP (mg/L)	2.25 (0.30–169.00)		
MPV (fL)	10.50 (8.30– 12.50)		
Neutrophil/lymphocyte ratio	1.90 (0.67– 6.56)		
Platelet/lymphocyte ratio	122.05(50.7–373.8)		
Activity Scores, median (min - max)			
BSAS	10 (0–54)		
BDCAF	2 (0–6)		

T test was used for parametric data.

In four patients, colchicine was ceased due to side effects. Azatioprin was the mostly commonly used (22.22%) disease modifying antirheumatic drug. In one patient, cyclophosphamide was used due to the acute thrombosis of superior vena-cava. Adalimumab was the only preferred biological disease-modifying antirheumatic drug in our patients, which was used for recurrent panuveitis and refractory gastrointestinal involvement. 

### 3.2. Association of serum HMGB- 1 level with BD manifestations

We observed that in patients who had genital ulcers and EN-like lesions in the past four weeks prior to recruitment had significantly higher levels of HMGB- 1. (p = 0.041 and p < 0.001) (Table 2). 

**Table 2:** Comparison of serum HMGB- 1 levels in BD patients with and without clinical involvement in the last four weeks.

Current clinical findings (n)	HMGB- 1 level median (min - max)	p value
Oral ulcer		0.218
(+) (n = 60)	44.03 (0–221.33)	
(-) (n = 30)	39.11 (0–103.91)	
Genital ulcer		0.041*
(+) (n = 7)	47.11 (43.26–221.33)	
(-) (n = 83)	42.50 (0–103.91)	
EN-like lesion		< 0.001*
(+) (n = 14)	51.83 (27.90–75.25)	
(-) (n = 76)	39.85 (0–221.33)	
Papulopustular eruption		0.216
(+) (n = 26)	45.17 (5.46–62.42)	
(-) (n = 64)	41.36 (0–221.33)	
Arthralgia/arthritis		0.723
(+) (n = 57)	43.26 (0–221.33)	
(-) (n = 33)	42.88 (0–103.91)	
Major organ involvement		0.314
(+) (n = 4)	44.3. (35.05–47.89)	
(-) (n = 86)	43.26 (0–221.33)	

(+): present; (-): absent

No statistically significant difference was found between patients with and without vascular, ocular or MOI involvements (p = 0.552, p = 0.329 and p = 0.212, respectively) (Table 3). 

**Table 3:** Comparison of serum HMGB- 1 levels in BD patients with and without eye, vascular and MOI involvement since the disease onset.

	HMGB- 1 level median (min - max)	p value
Eye involvement		0.329
(+) (n = 28)	45.18 (5.46–58.29)	
(-) (n = 62)	41.37 (0–221.33)	
Vascular involvement		0.552
(+) (n = 27)	45.56 (5.46–103.91)	
(-) (n = 63)	42.5 (0–221.33)	
Major organ involvement		0.212
(+) (n = 47)	44.79 (5.46–103.91)	
(-) (n = 43)	37.15 (0–221.33)	

Mann–Whitney U test was used.

### 3.3. Association of serum HMGB- 1 level with disease activity scores

There was weak but positive correlation between serum HMGB- 1 levels, BSAS and BDCAF scores (r = 0.24, *p *= 0.022 and r = 0.24, *p *= 0.026, respectively). Serum HMGB- 1 level of active BD patients (n = 52) was significantly higher than inactive patients (n = 38) (median 45.17 and 27.97 pg/mL respectively; *p *= 0.035).

## 4. Discussion 

HMGB- 1 is a nonhistone protein that can be found both inside and outside the cell and has different functions by its location [26]. It regulates transcription and autophagy when it is bound with DNA within the cell [26]. During cell activation, death or damage; it is released outside of the cell, acting as an alarmin or Damage Associated Molecular Pattern (DAMP) to activate the innate immune system [27]. Due to its interaction with chemokines, cytokines and Pathogen Associated Molecular Pattern (PAMP), HMGB- 1 is considered to play a role in the pathogenesis of inflammatory and autoimmune diseases [28].

In the literature, there are studies showing that serum HMGB- 1 levels are higher in patients with chronic systemic inflammatory diseases when compared to HC [12, 29–31]. Taniguchi et al. [32] measured the HMGB- 1 concentrations in sera and synovial fluids of 30 rheumatoid arthritis (RA) and 30 osteoarthritis patients and found statistically significant higher concentrations of HMGB- 1 in both sera and synovial fluids of RA patients. Goldstein et al. [29] also showed that serum HMGB- 1 levels were higher in RA patients compared to HC and that it correlated significantly with disease activity score of 28 joints. In the study with 147 ankylosing spondylitis (AS) patients, serum HMGB- 1 level of AS patients was significantly higher than in HC and it was correlated positively with AS disease activity indices such as Bath Ankylosing Spondylitis Disease activity index (BASDAI), Bath Ankylosing spondylitis Functional index (BASFI), Ankylosing spondylitis disease activity score-ESR (ASDAS-ESR), ASDAS-CRP, ESR, and CRP [31]. A study conducted with 169 Granulomatosis with polyangiitis (GPA) patients in Germany [33] demonstrated higher serum HMGB- 1 levels in GPA in comparison to HC and also in this study, higher serum HMGB- 1 levels were found in patients with more dominant granulomatous manifestations. To the best of our knowledge, there are only two studies in the literature evaluating the serum HMGB- 1 levels in BD patients. 

In our study, serum HMGB- 1 level was found to be significantly higher in BD patients than in HC. The results of the other two studies evaluating the serum HMGB- 1 levels in BD patients support our results [24,34]. Hyperfunction of neutrophils, vascular injury and autoimmune responses are involved in the pathogenesis of BD [14]. Innate immune system plays a critical role in the initiation and aggregation of inflammation in BD [34]. It was demonstrated that polymorphonuclear neutrophils adhere to endothelial cells and migrate to inflamed areas, which showed the central role of leucocytes in the pathogenesis of BD. In BD, there is not only enhanced neutrophil chemotaxis, but also the neutrophils are overreactive, which was proved by the increased production of superoxide and lysosomal enzymes. Alarmins are released as signaling molecules after trauma, infection or injury [35]. When the pattern recognition receptors of alarmins bind with pathogen associated molecular pattern molecules, the immune system is activated, proinflammatory cytokines are released and inflammation is triggered. HMGB- 1 acts as an alarmin in several tissues and activates the innate immune system [36]. As a result, serum HMGB- 1 level is considered to increase in BD as HMGB- 1 is a strong stimulator of the innate immune system [34]. De Souza et al. [24] found higher serum HMGB- 1 levels in the BD group (n = 26) than in the HC group (n = 20), but they did not show a relationship between BDCAF scores and serum HMGB- 1 levels (*p *= 0.339). In our study, though, we used both BSAS and BDCAF to evaluate disease activity which are reliable, validated and frequently used indices, and we found statistically significant but weakly positive correlations between these scores and serum HMGB- 1 levels. The higher number of patients in our study might have led to a significant relationship between activity scores and serum HMGB- 1 level. In addition, de Souza et al. used only BDCAF, but we used not only BDCAF but also BSAS, which is a newer activity measure of BD. To the best of our knowledge, there is no study in the literature that has evaluated the relationship between BSAS and HMGB- 1 level. As we found significantly positive correlations in both indices, this may indicate our results being more accurate and reliable. 

In some studies, patients with BDCAF score ≥ 2 were considered as “active” [24,25]. We also considered patients with BDCAF ≥ 2 as active in our study and examined the serum HMGB- 1 levels of active and inactive (BDCAF < 2) patients. We found significantly higher HMGB- 1 levels in active patients. Serum levels of the cytokines involved in neutrophils recruitment and activation such as CXCL8 and granulocyte-colony stimulating factor were shown to be increased in active BD [16]. This result shows that in active BD patients neutrophil activation is augmented which can explain the elevated levels of serum HMGB- 1 level in active patients. De Souza et al. [24] also considered patients with BDCAF score ≥ 2 as active, but found no significant difference between the serum HMGB- 1 levels of active (n = 13) and inactive (n = 13) patients. In the study conducted with 42 BD patients and 22 HC in Korea [34], a higher serum HMGB- 1 level in the BD group was found, and, even though, higher extracellular HMGB- 1 expression in active patients (n = 25) compared to inactive patients (n = 17) was shown, it was not statistically significant. This study has defined active disease as follows: having at least three of the following symptoms due to the worsening of clinical findings during the study: oral ulcer or stomatitis, genital ulcer, anterior iridocyclitis, posterior vasculitis or panuveitis, cutaneous symptoms, or pathergy positivity [34]. In our study, the correlation of serum HMGB- 1 level with other inflammatory markers such as ESR and CRP were also examined, but no significant correlation was found. Similarly, Ahn et al. [34] found no significant correlation between serum HMGB- 1 level, ESR, CRP and leukocyte count.

In our study, we also evaluated the association between serum HMGB- 1 level and clinical findings of BD patients. While mucocutaneous and joint involvements were evaluated according to their presence in the last four weeks, MOI (i.e. the presence of at least one involvement of the eye, vascular, CNS, and GIS) was evaluated as both by their presence in the last four weeks of recruitment and since the onset of the disease as the number of patients with current MOI was small in our study. However, we found no association between serum HMGB- 1 level and the presence of vascular, ocular and MOI as indicators of disease severity. On the other hand, significantly higher serum HMGB- 1 levels were found only in patients with genital ulcer and EN-like lesions. De Souza et al. [24] found no significant correlation between serum HMGB- 1 levels and both mucocutaneous and MOI (i.e., eye, neuro- and vasculo-Behcet’s diseases). Ahn et al. [34] showed statistically significant higher extracellular HMGB- 1 expression in patients with intestinal involvement than in those without intestinal involvement but could not found any association between other clinical findings. RAGE, a transmembrane protein belonging to the Ig superfamily, was the first receptor described for HMGB- 1 [4]. RAGE is expressed at low levels in normal tissues and vasculature but is upregulated at sites where its ligands accumulate causing neutrophil chemotaxis. Binding of RAGE by its ligands increases the permeability of the vascular endothelium and enhances the expression of adhesion molecules such as VCAM- 1 and proinflammatory cytokines like procoagulant tissue factor and or interleukin- 6 [37]. This is why it is thought that HMGB- 1 plays a role in vascular involvement in BD [33]. Orogenital ulcerations and EN-like lesions are examples of direct injury to vessel wall in BD [34]. In the study of Demirkesen et al [38], a neutrophilic vasculitis was shown in almost half of the nodular lesions of BD, and predominantly neutrophil infiltration was statistically important parameter proving the BD lesion. This may explain the high serum HMGB- 1 levels in our patients with genital ulcers and EN-like lesions. In this study, the number of patients with MOI in the last four weeks of recruitment was small, it might have affected our results. Therefore, the correlation between MOI and serum HMGB- 1 levels should be examined in further studies conducted with larger number of patients.

This study has some limitations. First, this was a cross-sectional study, which included only BD and HC. Association of serum HMGB- 1 level with BD could be interpreted more preciously if other inflammatory diseases were included as control group. Even if we found a correlation between disease activity indices and serum HMGB- 1 level, a prospective study should be designed to evaluate the change in serum HMGB- 1 level after appropriate disease control in active patients. Secondly, as the number of patients with current MOI was small, examining serum HMGB- 1 level with larger number of active patients may be useful in clarifying the role of HMGB- 1 in BD pathogenesis. In addition, examining HMGB- 1 levels at the tissue level may also be useful in explaining the correlation between HMGB- 1 and BD. For this purpose, it can be suggested to study HMGB- 1 levels, in biopsy samples from active mucocutaneous lesions and synovial fluid aspirates in patients with synovial inflammation secondary to BD. Lastly, in order to investigate the correlation of HMGB- 1 with other proinflammatory cytokines involved in BD pathogenesis, these cytokine levels in the serum can also be examined simultaneously.

## 5. Conclusion 

In this study, higher serum HMGB- 1 level was found in BD patients than in HC. Unlike other studies, this study also showed that serum HMGB- 1 levels in BD patients were positively correlated with BSAS and BDCAF scores. These results suggest that HMGB- 1 may have a role in BD development and can be used to monitor disease activity. These results should be supported by further studies that include higher number of patients and patients with other inflammatory diseases in order to clarify the association between HMGB- 1 and BD.

## Funding

Our study was funded by the Scientific Research Projects Unit of Ankara University (Project number: 18L0230003).

## Informed consent

The study was conducted according to the Declaration of Helsinki , and written consent was received from the patients. The study protocol was approved by Ankara University Faculty of Medicine Ethics Board (Approval Number: 12-724-17).
